# Effect of Different Interstocks on Fruit Quality, Amino Acids, and Antioxidant Capacity in ‘Yuanxiaochun’ Citrus

**DOI:** 10.3390/antiox14101149

**Published:** 2025-09-23

**Authors:** Tie Wang, Guochao Sun, Siya He, Ling Liao, Bo Xiong, Zhihui Wang

**Affiliations:** College of Horticulture, Sichuan Agricultural University, Chengdu 611130, China; wangtie@stu.sicau.edu.cn (T.W.); 71203@sicau.edu.cn (G.S.); hesiya@sicau.edu.cn (S.H.); 14622@sicau.edu.cn (L.L.); xiong-bo1221@sicau.edu.cn (B.X.)

**Keywords:** interstocks, fruit quality, citrus, sugar and acid components, amino acids

## Abstract

High grafting is a widely recognized technique for varietal renewal in aging citrus orchards. However, following high grafting, a specific ‘rootstock-interstock-scion’ combination is formed, yet the influence of interstock on scion fruit quality remains insufficiently explored. To address this gap, we conducted experiments by grafting ‘Yuanxiaochun’ ((*Citrus unshiu* Marcov × *Citrus sinensis* Osbeck) × (*Citrus reticulata* × *Citrus paradisi*)) onto three distinct interstocks (‘Yuanxiaochun’/‘Harumi’/‘*Trifoliate orange*’ (CJ), ‘Yuanxiaochun’/‘Ponkan’/‘*Trifoliate orange*’ (PG), ‘Yuanxiaochun’/‘Marumi Kumquat’/‘*Trifoliate orange*’ (JJ)), with ‘Yuanxiaochun’/‘*Trifoliate orange*’ used as a control (CK), and comprehensively evaluated their impact on fruit quality of ‘Yuanxiaochun’. Our research results show that interstock significantly increased the total soluble solids (TSSs) content of fruit. Additionally, interstocks also significantly increased the organic acid content in the fruit, particularly citric acid, which was on average 2.90 mg·g^−1^ FW higher than CK. In terms of fruit flavor, interstocks significantly reduced the sugar/acid ratio and the sweetness/total acid ratio. However, CJ and PG showed markedly higher sweetness levels. Furthermore, interstocks led to a marked increase in both total amino acid content and flavor-active amino acid content in the fruit. Taste active values of γ-aminobutyric acid, asparagine, aspartic acid, glutamic acid, and arginine were all greater than 1, indicating a significant contribution to the fruit flavor. Moreover, interstocks increased the total flavonoid and phenol content in the fruit, thereby affecting its overall antioxidant capacity. These findings provide valuable and systematic insights for high grafting and variety renewal in citrus production.

## 1. Introduction

Citrus is one of the world’s most important cash crops, valued for its unique nutritional and medicinal properties [1]. In China, Sichuan stands out as one of the most suitable ecological zones for cultivating late-maturing hybrid mandarin oranges, accounting for over half of the country’s planting area and production [2]. In the advancement of Sichuan’s late-maturing citrus industry, to align with market trends, a large number of exceptional and novel late-maturing citrus varieties have been cultivated or introduced, with high grafting technology being employed to update the varieties. High grafting makes full use of the strong root system, trunk, and abundant nutrients of the original plant, allowing for rapid crown formation and early fruiting. It plays a significant role in the transformation of orchard varieties, achieving the regionalization of superior cultivars, and improving both the yield and quality of fruits [3]. However, some studies have indicated that interstocks may have a negative impact on scions, such as causing graft incompatibility [4] or delaying flowering [5]. While this technique allowed for the rapid development of new varieties, it has also brought forth a novel rootstock−interstock (interstock is a section of the trunk located between the rootstock and the scion [6]). Studies have shown that interstocks exert significant influence on scion traits such as growth characteristics [7], stress tolerance [8], and fruit quality [9], among other factors.

Among these influencing factors, the impact of interstock on fruit quality stands out as a key point. In citrus research, it has been observed that ‘Ponkan’ (*Citrus reticulata* Blanco) as an interstock significantly increased fruit total soluble solids (TSSs) and decreased the titratable acid (TA) content of the scion ‘Harumi’ (*Citrus reticulata* × (*Citrus reticulata* × *Citrus sinensis*)) [10]. Similar findings were reported in apple cultivation, where the interstock M26 was found to enhance the accumulation of TSS and soluble sugars (SSs) in scion fruits [9]. Previous studies have highlighted that SSs and organic acids are primary factors influencing fruit flavor [11], and alterations in sugar/acid attributes mediated by the interstock could contribute to enhancing fruit flavor quality. However, it is important to note that flavor is a complex trait, intricately linked not only to sugars and organic acids but also to amino acids and various secondary metabolites [12]. Recent research has found that amino acids exist in the form of free amino acids (FAAs) and bound amino acids (BAAs) [13]. BAAs have less impact on food flavor as they are not degraded immediately after ingestion, making FAAs the main type of amino acid that affects fruit flavor quality. Our prior research also substantiated a significant influence of the rootstock on the amino acids in scion fruits [14]. Nonetheless, investigations into whether the interstock impacts the accumulation of amino acids in citrus fruits and thus influences fruit flavor have yet to be reported.

In addition to fruit flavor, researchers have also examined the influence of rootstock on the antioxidant capacity of the scion. For example, the antioxidant capacity of the peel in the ‘Satsuma mandarin’ (*Citrus unshiu*)/‘Flying dragon’ (*Citrus trifoliata* L.) combination was significantly higher than that of ‘Satsuma mandarin’/‘*Trifoliate orange*’ (*Poncirus trifoliata* (L.) Raf.), particularly in terms of hesperidin, total flavonoid, total phenol, and DPPH values [15]. Similarly, other citrus studies have demonstrated that certain rootstocks can enhance the antioxidant capacity of fruit juice [16]. However, some researchers have also noted that despite their substantial impact on the fruit vitamin C (Vc) of ‘Tango’ (*Citrus reticulata*, Blanco), rootstocks do not significantly affect the overall antioxidant capacity [17]. These findings indicate that the effect of rootstock on the antioxidant capacity of scion fruits is influenced by the specific rootstock and scion combination.

To investigate the impact of interstocks on citrus scions, we selected three citrus varieties from the Sichuan region, which are likely to be widely used for top grafting in the future, as interstock materials based on industry needs. We used the late-maturing, high-quality citrus variety ‘Yuanshaochun’ ((*Citrus unshiu* Marcov × *Citrus sinensis* Osbeck) × (*Citrus reticulata* × *Citrus paradisi*)) as scions to comprehensively investigate the effects of different interstocks on the conventional fruit quality, sugar/acid components, amino acid composition, phenolic compounds, and total antioxidant capacity of the scions. The results of this study will provide a comprehensive reference for understanding the impact of interstocks on citrus fruit quality and offer valuable insights for the industry’s top grafting practices.

## 2. Materials and Methods

### 2.1. Plant Material and Experimental Location

From the grafted seedlings cultivated previously [2], with ‘Yuanxiaochun’ citrus as scion (the scion grafting branches were taken from the same mother tree), 2-year-old ‘Harumi’ (*Citrus reticulata* × (*Citrus reticulata* × *Citrus sinensis*)), ‘Ponkan’ (*Citrus reticulata* Blanco cv. Ponkan), and ‘Marumi Kumquat’ (*Fortunella japonica* (Thunb.) Swing.) were used as interstock and grafted on *‘Trifoliate orange’* (*Poncirus trifoliata* (L.) Raf.). The diameter and length of the interstock were 0.6–0.8 and 10 cm, respectively. The length of the rootstock was 5 cm. Nine specimens with medium growth potential and no apparent pests or diseases per treatment were selected as test subjects (not pruned). These were transplanted with a spacing of 2 m × 2 m. Standard horticultural management practices were employed for all plantings. Each plot accommodated three plants, with three replications established. Specifically, the scion for the grafted seedling was ‘Yuanxiaochun’ and the rootstock was ‘*Trifoliate orange*’. The interstocks of the grafted seedlings included ‘Harumi’, ‘Ponkan’, and ‘Marumi Kumquat’. The control check consisted of ‘Yuanxiaochun’ grafted onto ‘*Trifoliate orange*’ (CK). Each grafting combination was denoted by CJ, PG, JJ, and CK, respectively (Table 1).

The experimental site, situated in Zhuangjia Village, Hongyuan Township, Jianyang City, Sichuan Province (longitude: 104°; latitude: 30°), experiences a subtropical monsoon climate. It boasts an average annual temperature of 17 °C, annual precipitation averaging 874 mm, and an approximate annual frost-free period of 311 days.

### 2.2. Test Sample Collection and Processing

Each treatment consisted of three trees with a similar fruit set, grouped into a small plot, and each treatment had three replicates. Fruits were harvested at the ‘Yuanxiaochun’ ripening stage (13 January 2024), following the harvest time recommended by previous studies [18]. Five fruits free of pests, diseases and mechanical damage, with proper fruit shape and uniform size, were selected from each of the five directions in the outer and middle layers of the canopy of each tree: east, south, west, north and center. A total of 45 fruits were collected per treatment. The harvested fruit was quickly placed in foam boxes containing ice packs and transported back to the lab. After washing the fruit surfaces with distilled water, excess moisture was wiped off using laboratory paper. Subsequently, measurements were carried out of single fruit weight, and longitudinal and transverse diameters. Following the assessment of these basic parameters, the fruit peel and pulp were separated. The pulp was rapidly chopped and homogenized, treated with liquid nitrogen, and then stored at −80 °C for later use.

### 2.3. Fruit Sensory Evaluation

Sensory evaluation was conducted, drawing upon previous research methods with slight adjustments [19]. A team of twelve professionals, comprising six males and six females, experienced in assessing citrus fruit quality, was assembled. Prior to the evaluation, the evaluators underwent brief training sessions to comprehensively explain the evaluation methodology and objectives. The evaluation criteria primarily encompassed sweetness, sourness, aroma, bitterness, and off-flavors, focusing on the flavor profile of citrus fruits. Each evaluation criterion had a maximum score of 10 and a minimum of 0, with higher scores indicative of more pronounced flavors.

### 2.4. Determination of Standardized Quality Parameters

Single fruit weight was measured using an electronic balance (AL204, METTLER, Greifensee, Switzerland). The longitudinal and transverse diameters of the fruit were measured with vernier calipers, and the length/diameter ratio was used as the fruit shape index (in citrus fruits, a fruit shape index of less than or equal to 0.80 typically indicates a flattened-round shape; an index between 0.81 and 0.90 corresponds to a round or near-round shape; and an index greater than 0.91 is generally associated with an oval shape.) [20]. TSSs and TA were measured with a sugar/acid integrated machine (Pocket PAL-BXIACID1, ATAGO, Tokyo, Japan) [21]. Vc content was determined by 2,6-dichlorophenol indophenol titration [22], and the pH of the fruits was measured following the method of Zahir et al. using a pH meter (pH828, SMARTSENSOR, Shanghai, China) [23]. pH measurement ranged from 0 to 14, with a resolution of 0.01 and an accuracy of ±0.01.

The free amino acid content was estimated using the ninhydrin method, and a standard curve was constructed using L-leucine as the reference compound [24]. A pulp sample weighing 1.0 g was accurately measured and placed in a mortar and pestle, followed by the addition of 5.0 mL of 10% acetic acid for extraction. Next, 1.0 mL of the filtered sample was pipetted into a 20 mL dry stoppered graduated test tube, and 1.0 mL of ammonia-free distilled water and other reaction reagents was added. After the completion of the reaction, the absorbance value of the solution was measured at 570 nm. The results obtained were expressed as milligrams of ammoniacal nitrogen per 100 g of fruit pulp.

The total flavonoid content was determined using the aluminum chloride colorimetric method [25]. In a 10 mL centrifuge tube, 0.5 mL of fruit pulp extract was taken separately, reaction reagents including 0.7 mL of pure water and 0.2 mL of 5% NaNO_2_ were added and then fixed to 5 mL, the absorbance value of the solution to be tested was measured at 500 nm with a spectrophotometer, and the results were expressed as Rutin Equivalent (RE).

The total phenol content was determined by the Folin–Ciocalteu colorimetric method [26]. Initially, 250 μL of pulp extract was pipetted into a 5 mL centrifuge tube, followed by the addition of 750 μL distilled water and 1.0 mL of Folin–Ciocalteu reagent at a 1:1 dilution. The mixture was vortexed, left in the dark for 5 min, and then 1 mL of 5% Na_2_CO_3_ solution was added. After thorough mixing, the solution was allowed to react for 60 min away from light. Finally, the absorbance values of the solutions were measured at 650 nm using the extraction solvent as a control, and the results were expressed as gallic acid equivalents (GAEs).

### 2.5. Determination of Sugar/Acid Components of Fruit Pulp and Calculation of Sugar/Acid Ratio

Fructose, glucose, and sucrose contents were determined using high-performance liquid chromatography (HPLC) [27]. Initially, 2.0 g of fruit pulp was accurately weighed, followed by the addition of 4 mL of distilled water, which was then thoroughly shaken. The mixture was held in a water bath at 80 °C for 15 min, followed by natural cooling and subsequent centrifugation at 4 °C and 9000 rpm for 15 min. The supernatant was collected, and the residue was subjected to another extraction with 4 mL of distilled water. The resulting supernatant was combined with the previous one and adjusted to a total volume of 10 mL. Next, 1.50 mL of the extract was drawn with a disposable syringe and filtered through a 0.45 μm aqueous filtration membrane into the injection bottle for analysis using the chromatography machine.

Organic acid components were determined by HPLC with reference to previous methods [27]. The sample was weighed as 0.5 g of mixed fruit pulp, fully mixed with 3 mL of pre-cooled 0.2% metaphosphoric acid and volume-adjusted to 6 mL, and centrifuged at 12,000 rpm/min at 4 °C for 15 min, and 1.5 mL of the extracted sample solution was extracted with a disposable syringe and filtered through 0.22 μm aqueous filtration membrane into the injection vial for measurement on the machine. Sugar/acid ratio = total sugar content (sum of sugar components)/total acid content (sum of acid components).

### 2.6. Fruit Sweetness Calculation and Fruit Flavor Evaluation

Fruit sweetness value calculation was conducted following the method outlined by Wang et al. [28], whereby the sweetness value was determined as follows: sweetness = fructose content × 175 + glucose content × 70 + sucrose content × 100. Fruit flavor evaluation was performed in accordance with the approach described by Zhao et al. [29], where the flavor value was expressed as the ratio of the sweetness value to the total acid content.

### 2.7. Determination and Evaluation of Fruit Amino Acid Components

Amino acid components were determined with reference to the chromatographic mass spectrometric detection method described by previous authors [30,31], and the assay was measured by Sichuan Panomix Biotechnology Co., Suzhou, China. Weigh 50 mg of the sample into a 2 mL centrifuge tube, and accurately add 600 μL of a 10% formic acid-methanol solution in water (1:1, *v*/*v*). Add two steel beads and vortex for 30 s. Place the tube in a tissue grinder and grind at 55 Hz for 90 s. Centrifuge at 12,000 rpm, 4 °C for 5 min. Take 20 μL of the supernatant, dilute it 50 times by adding 980 μL of a 10% formic acid–methanol solution in water (1:1, *v*/*v*), and then vortex for 30 s. Take 100 μL of the supernatant and add 100 μL of 100 ng/mL Trp-d3 internal standard, vortex for 30 s. Filter the supernatant through a 0.22 μm filter membrane, and transfer the filtrate into a detection vial. Chromatographic–mass spectrometric detection conditions: ACQUITY UPLC^®^ BEH C18 Column (2.1 × 100 mm, 1.7 μm, Waters, Houston, TX, USA), The injection volume was 5 μL, the column temperature was 35 °C, and the mobile phase A was 50% methanol in water (containing 0.1% formic acid), B was 10% methanol water (containing 0.1% formic acid). The gradient elution conditions were 0–6.5 min, 90–70% B; 6.5–7 min, 70–0% B; 7–14 min, 0% B: 14–14.5 min, 0–90% B; 14.5–17.5 min, 90% B. Flow rate 0~8.0 min, 0.3 mL/min; 8.0~17.5 min. 0.4 mL/min. Mass spectrum condition: Electrospray ionization (ESI) source, positive ionization mode. The ion source temperature was 500 °C, the ion source voltage was 5500 V, the collision gas was 4 psi, the curtain gas was 40 psi, and the atomizing gas and auxiliary gas were both 50 psi. Scans were performed using multiple reaction monitoring (MRM). The abbreviations and full names of the amino acid components are as follows: Gly: glycine; Ala: alanine; Val: valine; Leu: leucine; Ile: isoleucine; Phe: phenylalanine; Pro: proline; Trp: tryptophan; Ser: serine; Tyr: tyrosine; Met: methionine; Asp: aspartic acid; Asn: asparagine; Gln: glutamine; Glu: glutamic acid; Thr: threonine; Lys: lysine; Arg: arginine; His: histidine; GABA: γ-aminobutyric acid; Orn: ornithine; Hcy: homocysteine.

After the samples were assayed, each treated amino acid species was categorized according to the previous classification criteria [32] and further evaluated according to the sensory thresholds of different amino acids using the taste active value (TAV) [33,34].

### 2.8. Determination of Phenolic Components in Fruits

Fruit phenolic components were measured with reference to the method established by the group in the previous period [35], and the conclusions of previous studies were referred to characterize and quantify the main 10 phenols [36,37]. Thus, 0.5 g of mixed fruit pulp was accurately weighed into a 2 mL centrifuge tube, and 1.5 mL of extraction buffer was added and mixed thoroughly under light-avoidance conditions. After ultrasonication and centrifugation, 1.5 mL of the extract was withdrawn with a disposable syringe and filtered through a 0.45 μm microporous filter membrane into a feed bottle for measurement.

### 2.9. Determination of Total Antioxidant Capacity of Fruits

The total antioxidant capacity of the fruits was assessed using assay kits for the DPPH, ABTS, and FRAP methods, with all reagents procured from Shaanxi Puyinte Bioengineering Co., Shaanxi, China. To comprehensively and systematically evaluate the antioxidant activity of the pulp, we utilized the antioxidant potency composite (APC) index for assessment [38]. The formula for calculating the sample APC (%) is as follows:

Sample APC (%) = ((Sample DPPH/Maximum DPPH in all treatments) + (Sample ABTS/Maximum ABTS in all treatments) + (Sample FRAP/Maximum FRAP in all treatments))/3 × 100.

### 2.10. Statistical Analysis

Statistical significance tests were analyzed using ANOVA in SPSS 23.0 (IBM, Armonk, NY, USA), with significance set at *p* ≤ 0.05. Graphs were created using Excel 2016 (Microsoft, Redmond, Washington, DC, USA) and Origin 2021 (OriginLab Corporation, Northampton, MA, USA).

## 3. Results

### 3.1. Effects of Different Interstocks on Standard Quality Parameters of ‘Yuanxiaochun’ Fruit

**Figure 1:**
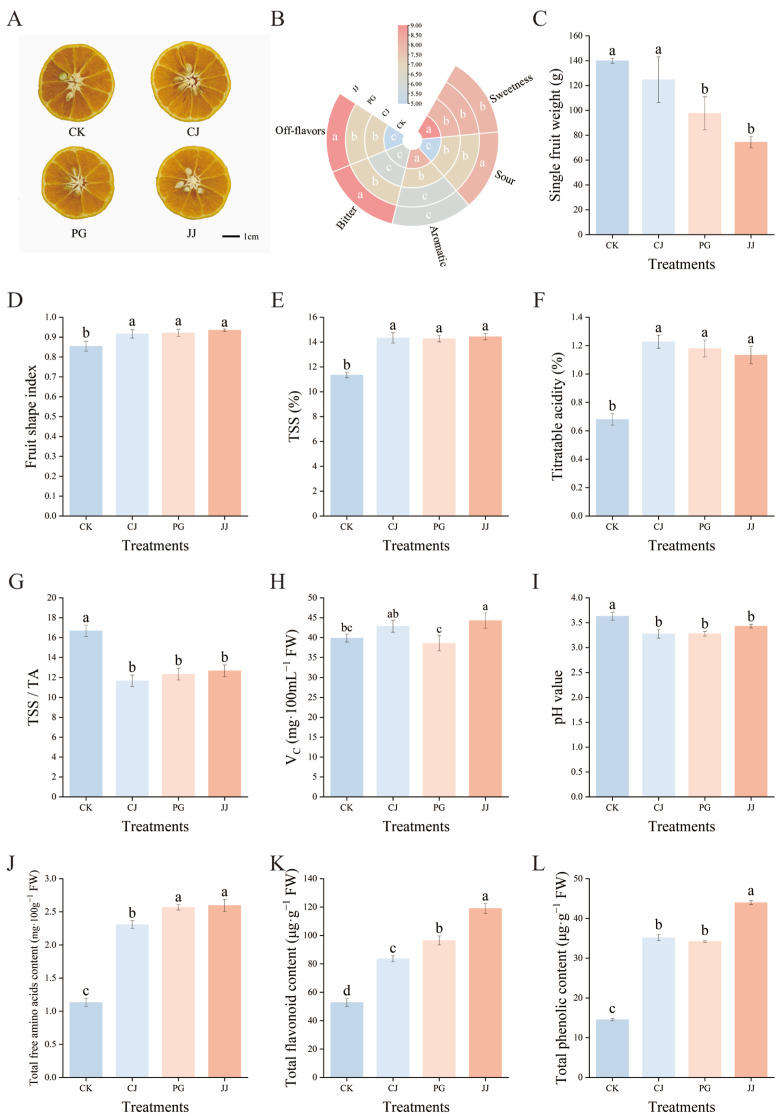
Effects of different interstocks on fruit quality of ‘Yuanxiaochun’. (**A**) Fruit cross-section pictures, (**B**) Fruit sensory evaluation chart, (**C**) Single fruit weight, (**D**) Fruit shape index, (**E**) TSSs, (**F**) TA, (**G**) TSS/TA, (**H**) Vc, (**I**) pH, (**J**) Total free amino acids, (**K**) Total flavonoid, (**L**) Total phenolic. The parameter values presented in each figure are indicated as mean ± standard error (n = 3). Different letters denote statistically differences between different treatments (Duncan’s test, *p* ≤ 0.05).

The impact of different interstocks on the standard quality parameters of ‘Yuanxiaochun’ exhibited significant differences (Figure 1). Cross-sectional images of the fruit showed that all graft combinations contained seeds (Figure 1A). Sensory evaluation indicated that the sweetness was most pronounced in the CK, CJ, and PG treatments, with the CK and CJ treatments exhibiting the strongest aroma. In contrast, the JJ treatment resulted in higher levels of bitterness, off-flavors, and acidity in fruits (Figure 1B). The individual fruit weight of the CK and CJ treatments significantly exceeded that of the other treatments, with the JJ treatment recording the lowest average single fruit weight at 74.43 g, marking a 46.80% decrease compared to CK (Figure 1C). The fruit shape index of the four treatments was lower than 0.9 only for CK, which showed a round shape, while the fruit shape index of the interstock grafted fruits ranged from 0.9 to 1.0 and showed an oval shape (Figure 1D).

Regarding the quality of the internal fruit, there were significant differences between CK and the interstock treatments. The CK treatment exhibited low TSSs, low TA, and a higher TSS/TA ratio, while the three interstock treatments showed opposite trends, with no significant differences observed (Figure 1E–G). In terms of V_C_ content, the JJ treatment had a significantly higher level, measuring 44.27 mg·100 mL^−1^ FW, which was notably greater than that of the CK and PG treatments (Figure 1H). Juice pH is not only an important indicator for evaluating fruit acidity but also has a significant impact on flavor presentation [39,40]. In this study, pH values for all juice treatments ranged from 3.27 to 3.63, with CK juice having the highest pH at 3.63. The CJ, PG, and JJ treatments showed no significant differences among themselves but the values were significantly lower than those of the CK treatment (Figure 1I). Further analysis in conjunction with the TA results revealed an opposite trend between pH and TA (Figure 1F,I). Additionally, trends in total free amino acids, total flavonoids, and total phenol content of fruits were consistent across all treatments, with significantly lower levels in CK and significantly higher levels in JJ (Figure 1J–L). Comprehensive analysis indicates that interstock has a significant impact on the fruit quality of ‘Yuanxiaochun’.

### 3.2. Effects of Different Interstocks on Sugar and Acid Components and Flavor Indices of ‘Yuanxiaochun’ Fruits

#### 3.2.1. Sugar Components

**Figure 2:**
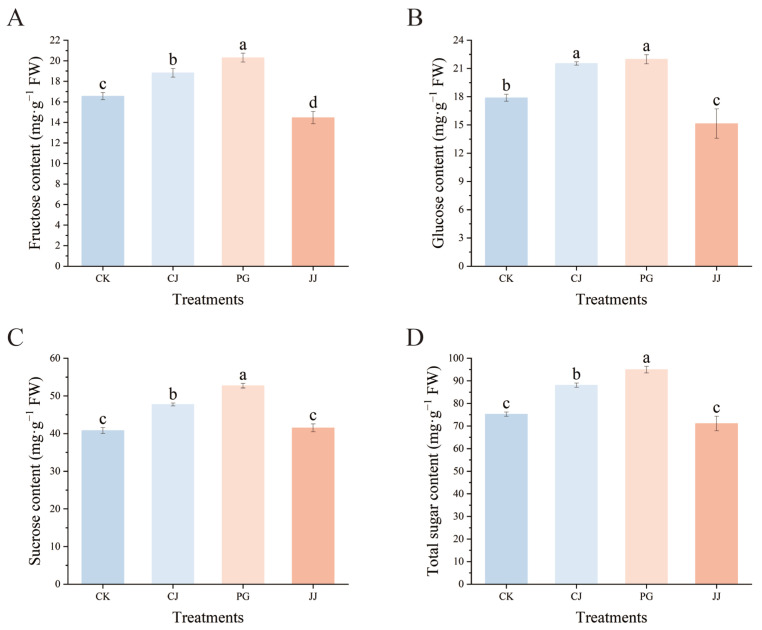
Effects of different interstocks on sugar components and total sugars of ‘Yuanxiaochun’ fruits. (**A**) Fructose. (**B**) Glucose. (**C**) Sucrose. (**D**) Total sugar. The parameter values presented in each figure are indicated as mean ± standard deviation (n = 3). Different letters denote statistically differences between different treatments (Duncan’s test, *p* ≤ 0.05).

Fructose, glucose, and sucrose were detected in the pulp of all four treatments by HPLC, with the highest sucrose content and a relatively low fructose content (Figure 2). Among the three sugars, the PG and CJ treatments had significantly higher levels of fructose, glucose, sucrose, and total sugar compared to the CK and JJ treatments. In terms of total sugar content, PG treatment had the highest level at 95.00 mg·g^−1^ FW, followed by the CJ treatment at 88.07 mg·g^−1^ FW, and the JJ treatment had the lowest at 71.13 mg·g^−1^ FW. Overall, the analysis indicates that both the PG and CJ treatments significantly enhance sugar accumulation in fruit compared to the CK treatment.

#### 3.2.2. Acid Components

**Figure 3:**
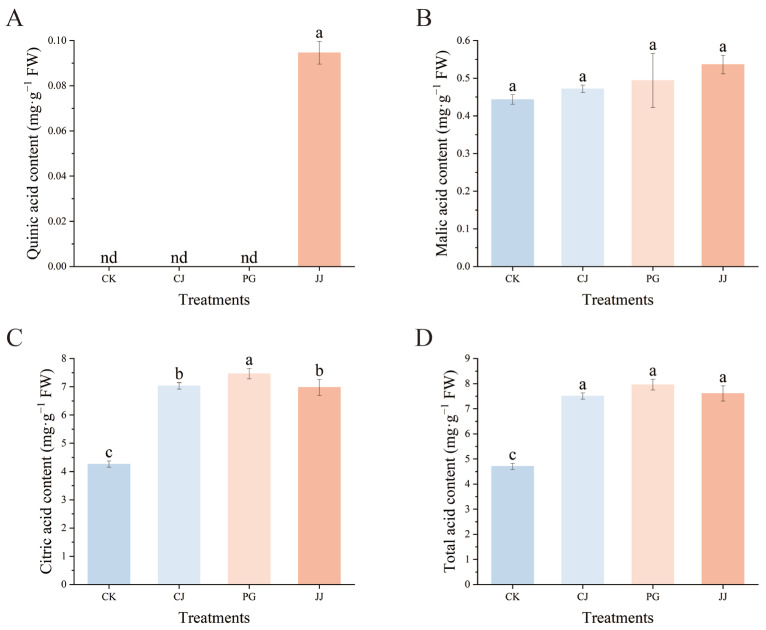
Effects of different interstocks on acid components and total acid of ‘Yuanxiaochun’ fruits. (**A**) Quinic acid. (**B**) Malic acid. (**C**) Citric acid. (**D**) Total acid. The parameter values presented in each figure are indicated as mean ± standard deviation (n = 3). Different letters denote statistically differences between different treatments (Duncan’s test, *p* ≤ 0.05).

The effect of different interstocks on the acid components of ‘Yuanxiaochun’ fruits is depicted in Figure 3. A total of three organic acids were detected in fruit pulp, with quinic acid being detected only in JJ pulp and its content being low at 0.09 mg·g^−1^ FW. In terms of malic acid content, the JJ treatment exhibited a higher level, while the CK treatment showed a lower level. However, the differences among the treatments were not significant. Trends in citric acid and malic acid content are inconsistent, with citric acid levels higher than malic acid. Specifically, the PG treatment showed the highest citric acid content at 7.46 mg·g^−1^ FW, while the CK treatment had significantly lower levels at 4.26 mg·g^−1^ FW. This indicates that using an interstock does not alter the primary pattern of organic acid accumulation in ‘Yuanxiaochun’ fruits, which mainly consists of citric acid. Additionally, this study found that the total acid content showed a trend consistent with citric acid. In summary, compared with the CK treatment, the other three interstocks significantly enhanced the accumulation of organic acids in ‘Yuanxiaochun’ fruits, especially citric acid.

#### 3.2.3. Sugar/Acid Ratio, Sweetness and Flavor

**Figure 4:**
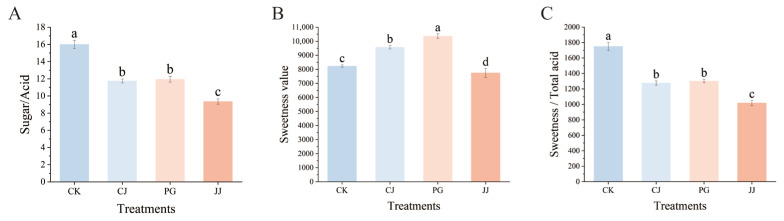
Effects of different interstocks on sugar/acid ratio, sweetness and flavor of ‘Yuanxiaochun’ fruits. (**A**) Sugar/acid ratio. (**B**) Sweetness. (**C**) Sweetness/total acid. The parameter values presented in each figure are indicated as mean ± standard deviation (n = 3). Different letters denote statistically differences between different treatments (Duncan’s test, *p* ≤ 0.05).

To comprehensively compare the effects of different interstocks on the fruit sugar/acid of ‘Yuanxiaochun’, we investigated the sugar/acid ratio. The results indicated that the CK treatment had a significantly higher sugar/acid ratio of 16.00. The differences between the PG treatment and the CJ treatment were not significant, but they were both significantly higher than those of the JJ treatment (Figure 4A). However, in the TSS/TA data, we found that the differences between the CJ, PG, and JJ treatments were not significant, which is inconsistent with the results of the sugar/acid ratio (Figure 1G).

To compare the reliability of the TSS/TA and sugar/acid ratio methods, we introduced a sweetness and flavor evaluation method for supplementary assessment (Figure 4B,C). The results indicated that the PG and CJ treatments were significantly sweeter, while JJ was significantly less sweet. Further evaluation of the flavor of each treatment showed complete agreement with the sugar/acid ratio data, with significantly higher flavor values for CK and significantly lower values for JJ. In summary, among the three interstocks, the CJ and PG treatments performed better. Additionally, there were differences among all the different fruit quality evaluation methods, but there was higher consistency between the sugar/acid ratio and flavor evaluation value, suggesting that it might be more suitable for citrus fruit quality evaluation.

### 3.3. Effect of Different Interstocks on Amino Acids in ‘Yuanxiaochun’ Fruits

**Figure 5:**
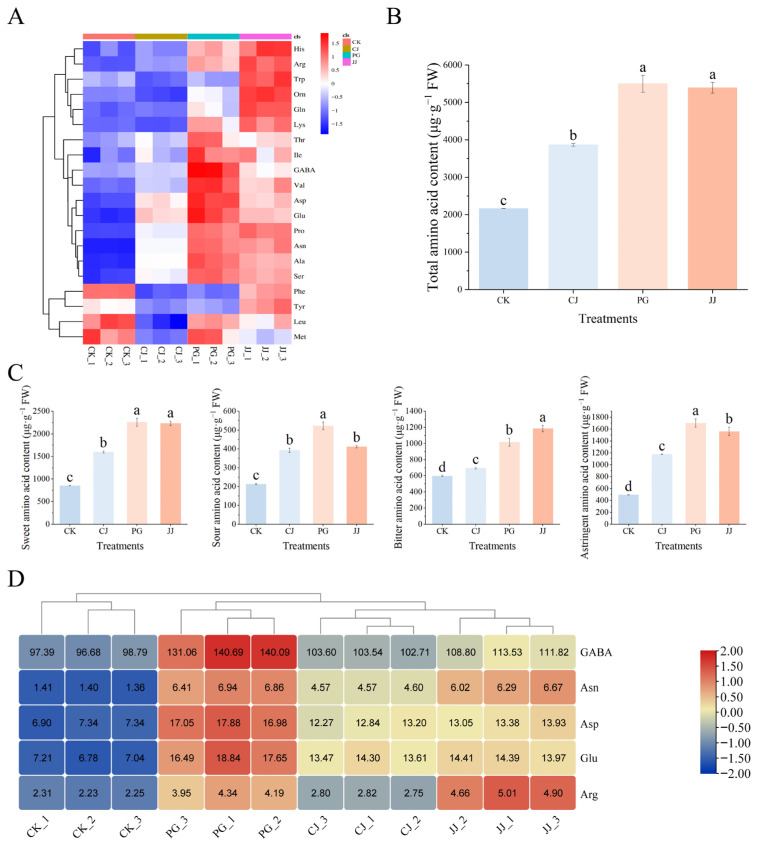
Effects of different interstocks on amino acid species and content of ‘Yuanxiaochun’ fruits. (**A**) Heat map of amino acid content (Gly and Hcy were not detected). (**B**) Total amino acid content. (**C**) Flavor amino acid content. (**D**) Amino acid statistics for TAV > 1. The parameter values presented in each figure are indicated as mean ± standard deviation (n = 3). Different letters denote statistically differences between different treatments (Duncan’s test, *p* ≤ 0.05).

The effect of different interstocks on the amino acid content in ‘Yuanxiaochun’ fruits is shown in Supplementary Table S1. A total of 20 out of 22 amino acids were detected, with the highest content of Pro, the lowest content of Met, Gly and Hcy not detected in any of the treatments. Among the treatments, PG and JJ pulp had a higher amino acid content, while that of the other treatments was lower. The total amino acid content analysis revealed that the PG and JJ treatments had significantly higher levels of amino acids in the fruit flesh, measuring 5500.35 μg·g^−1^ FW and 5393.26 μg·g^−1^ FW, respectively. In contrast, the CK treatment displayed a significantly lower total amino acid content at 2165.68 μg·g^−1^ FW (Figure 5B). Further categorization of amino acid components revealed that the total sweet amino acid content was higher and the sour amino acid content was lower in the pulp of all treatments. Among these, PG pulp had the highest total sweet amino acid, sour amino acid and astringent amino acid content, but JJ pulp had a significantly higher total bitter amino acid content (Figure 5C).

To clarify the specific contribution of these 20 amino acids to fruit quality, we evaluated each amino acid using TAV values and found that the TAV values of GABA, Asn, Asp, Glu, and Arg were all greater than 1, suggesting that these amino acids have a greater impact on fruit quality, especially GABA, which had a TAV value of more than 90 in all groups (Figure 5D). According to a previous study, GABA and Asn were found to be astringent, Asp and Glu were found to be sour, and Arg was found to be bitter [32]. In PG pulp, the content of all four substances except Arg was high, and it was hypothesized that the sour and astringent taste of PG pulp was closely related to GABA, Asn, Asp, and Glu. In addition, Arg TAV in the JJ treatment was higher than in other treatments, reaching 4.85. These findings underscore the critical role that these specific amino acids play in influencing the sensory attributes of ‘Yuanxiaochun’ fruits.

### 3.4. Effects of Different Interstocks on Phenolic Components and Antioxidant Capacity of ‘Yuanxiaochun’ Fruits

#### 3.4.1. Phenolic Components

**Figure 6:**
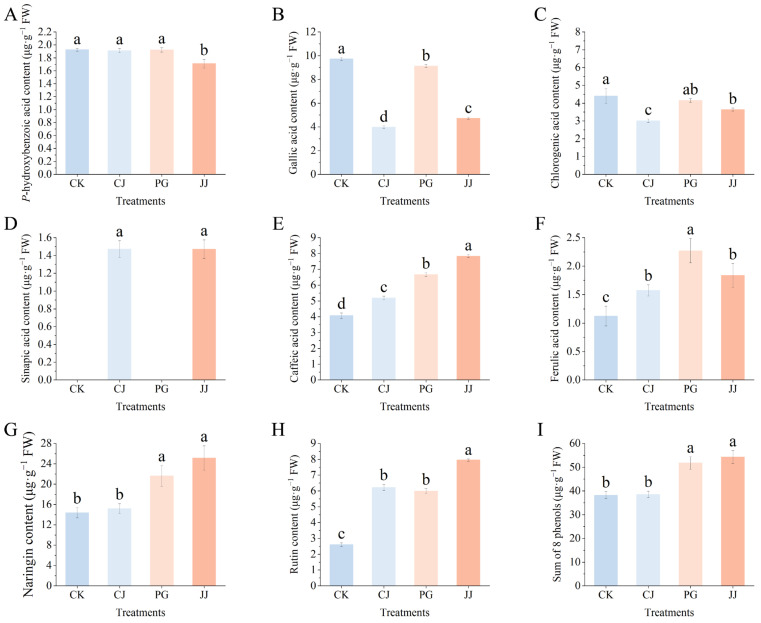
Effects of different interstocks on phenolic components of ‘Yuanxiaochun’ fruits. (**A**) *P*-hydroxybenzoic acid. (**B**) Gallic acid. (**C**) Chlorogenic acid. (**D**) Sinapic acid. (**E**) Caffeic acid. (**F**) Ferulic acid. (**G**) Naringin. (**H**) Rutin. (**I**) Sum of 8 phenols. Neohesperidin and naringenin were not detected. The parameter values presented in each figure are indicated as mean ± standard deviation (n = 3). Different letters denote statistically differences between different treatments (Duncan’s test, *p* ≤ 0.05).

In Figure 1K,L, we observed significant differences in the total flavonoid and total phenolic content of fruits under different treatments. To elucidate the specific phenolic constituents contributing to these variances, an analysis of ten phenolic compounds predominantly found in citrus was conducted. Notably, neohesperidin and naringenin were absent, while sinapic acid was only identified in CJ and JJ (Figure 6). Naringin demonstrated the highest concentration of the eight phenolic compounds examined, with the JJ treatment displaying the most significant accumulation of this compound. Conversely, the CK and CJ treatments showcased diminished levels of naringin. Furthermore, fruit flesh from the CJ treatment showed significantly lower levels of gallic acid and chlorogenic acid, while the content of sinapic acid was notably higher. In contrast, the JJ treatment not only had significantly elevated levels of naringin and sinapic acid, but also exhibited higher concentrations of caffeic acid and rutin. Notably, the CK treatment had the lowest rutin content, measuring only 2.60 μg·g^−1^ FW.

The comprehensive evaluation of the eight phenolic compounds demonstrated that the application of the JJ and PG treatments notably augmented the presence of phenolic substances in fruit pulp when compared to the CK treatment, with a specific emphasis on the enhancement in naringin levels. To summarize, diverse interstocks exhibit distinct effects on phenolic compound composition in ‘Yuanxiaochun’ fruits. The JJ and PG treatments facilitated increased levels of accumulation, while the CJ treatment did not exhibit marked differences from the CK treatment.

#### 3.4.2. Total Antioxidant Capacity

**Figure 7:**
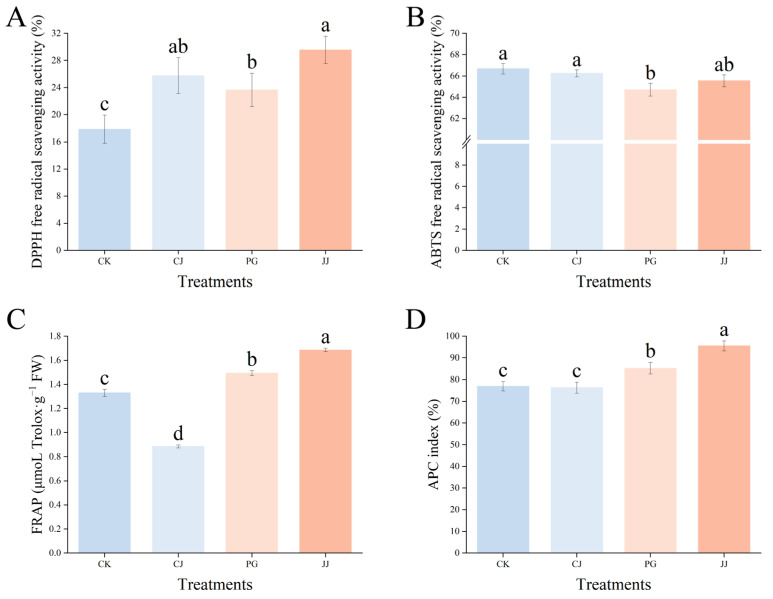
Effect of different interstocks on the total antioxidant capacity of ‘Yuanxiaochun’ fruits. (**A**) DPPH. (**B**) ABTS. (**C**) FRAP. (**D**) APC. The parameter values presented in each figure are indicated as mean ± standard deviation (n = 3). Different letters denote statistically differences between different treatments (Duncan’s test, *p* ≤ 0.05).

To comprehensively evaluate the total antioxidant level of various antioxidant substances, enzymes, and other components in the pulp of different treatments, we evaluated the total antioxidant capacity of fruits using DPPH, ABTS, and FRAP methods. As shown in Figure 7, the antioxidant activity of the same fruits determined by the three methods exhibited significant differences. In the DPPH assay, fruit flesh fromthe JJ treatment exhibited the highest antioxidant capacity at 29.56%, while the CK treatment showed the lowest antioxidant capacity at 17.86%. In ABTS, the differences between the three treatments were not significant except for the PG treatment, where pulp antioxidant capacity was slightly lower. In FRAP, the JJ treatment pulp had significantly higher antioxidant capacity, while CJ had the weakest, with 0.89 μmoL Trolox·g^−1^ FW and 1.69 μmoL Trolox·g^−1^ FW, respectively.

Further evaluation of the overall antioxidant capacity using the APC index revealed that the JJ treatment had the highest APC index at 95.56%, while the CK and CJ treatments had significantly lower values at 76.95% and 76.29%, respectively. This indicates that the antioxidant capacity of fruit flesh from the JJ treatment is the strongest, while that of the CK and CJ treatments is relatively weak. In summary, different interstocks exert different effects on the antioxidant capacity of ‘Yuanxiaochun’ fruits, and significant differences exist between the methods of measurement used.

#### 3.4.3. Correlation Analysis of Total Antioxidant Activity with Phenolics

**Figure 8:**
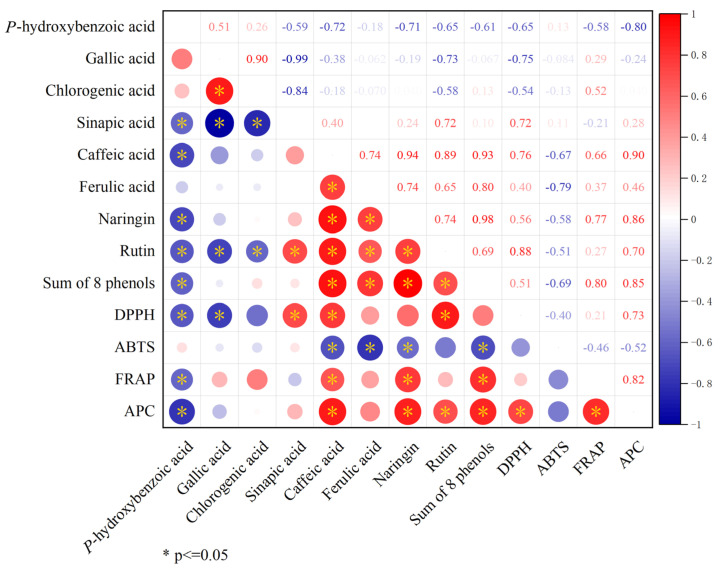
Correlation analysis of total antioxidant activity with phenolics.

Correlation analysis examined the relationship between phenolic compounds and total antioxidant activity. The results showed that the sum of eight phenols was significantly positively correlated with caffeic acid, ferulic acid, naringin, and rutin, and significantly negatively correlated with *p*-hydroxybenzoic acid. Further analysis revealed that DPPH, FRAP, and APC were all significantly positively correlated with caffeic acid. In addition, this study found that APC was significantly positively correlated with naringin, rutin, the sum of eight phenols, DPPH, and FRAP, but significantly negatively correlated with *p*-hydroxybenzoic acid (Figure 8).

## 4. Discussion

### 4.1. Interstocks Give Fruits Different Sweetness and Flavor by Affecting Their Sugar and Organic Acid Content

Interstocks, as a special section of rootstocks, are mostly found in the citrus industry as a result of variety renewal [2]. Grafting using interstocks results in rapid tree growth and early production. Current research indicates that citrus interstocks have a regulatory effect on fruit quality [6,10]. Our results reached a similar conclusion, showing that interstocks not only affect scion fruit size but also significantly impact TSSs, TA, Vc, and pH (Figure 1). Research on the influence of grafting on fruit size has been reported in both vegetables [41] and fruit trees [42]. Researchers have found that such changes may be related to grafting-induced alterations in metabolites and epigenetic regulation [43]. In our study, we found that the fruit size of ‘Yuanxiaochun’ grafted onto different interstocks exhibited significant differences. The specific reasons for these differences still require further investigation and exploration.

Further studies on fruit sugar/acid components revealed that the sugar/acid profiles of interstock and CK fruits were similar, both dominated by sucrose accumulation in sugar components and citric acid in organic acids, aligning with previous findings (Figure 2 and Figure 3) [27,44]. TSS/TA often serves as a fundamental index for flavor evaluation; higher TSS/TA ratios lead to a more intense flavor [45]. Our results show that the TSS/TA ratio in the CK treatment was significantly higher (17.0), while the ratios in the CJ, PG, and JJ treatments were significantly lower, with no significant differences between them. This is inconsistent with sensory evaluation results (Figure 1B), indicating that using only the TSS/TA ratio to evaluate flavor is limited.

To scientifically assess fruit quality, we introduced three comprehensive evaluation indices: the sugar/acid ratio, sweetness, and sweetness/total acid. We observed discrepancies among these indices compared to the TSS/TA trend (Figure 1G and Figure 4), speculating that this difference may arise because TSS is a comprehensive parameter incorporating materials such as fiber and minerals, in addition to sugar and acid [46]. Regarding sweetness, we noted a significantly higher value of 10,326.58 for PG, attributed to ‘Ponkan’ grafted ‘Yuanxiaochun’ fruits accumulating notably more fructose, glucose, sucrose, and total sugar directly (Figure 2). Similarly, our previous study identified ‘Ponkan’ as an interstock promoting sugar accumulation in scion fruits, possibly due to ‘Ponkan’ inducing the differential expression of genes such as *CINV2*, *FRK4*, and *HXK1* in scion fruits [10]. Furthermore, consistent results were observed between the sugar/acid ratio and the sweetness/total acid evaluation, both indicating CK as the highest and JJ as the lowest. This alignment with sensory evaluation results suggests that these two evaluation methods may be more suitable for interstock-mediated assessment of fruit sugar/acid quality, surpassing the efficacy of the TSS/TA evaluation method.

### 4.2. Interstock Affects Fruit Flavor by Regulating Amino Acid Content

Proteins, composed of amino acids, are indispensable and essential substances in human and animal nutrition [47]. In our study, we observed that ‘Yuanxiaochun’ grafted on different interstocks exhibited the highest Pro content and the lowest Met (Supplementary Table S1). This finding aligns with previous research, suggesting that interstocks do not alter the primary pattern of amino acid species accumulation in ‘Yuanxiaochun’ [48]. Further investigations unveiled significant differences in the content of homologous amino acids in ‘Yuanxiaochun’ fruits grafted on different interstocks, indicating that interstocks exert a significant influence on the accumulation of specific amino acids.

In the classification study of amino acid components, the TAV values for all treated fruits were lower than 1 in spite of the high content of sweet amino acids, implying that these amino acids did not contribute to the fruits’ sweetness (Figure 5C). Furthermore, our results revealed that the trend of sour amino acid content was essentially consistent with the total acid content of the fruits. Among these sour amino acids, Asp and Glu were also identified as having TAV >1. This suggests that sour amino acids, particularly Asp and Glu, could serve as auxiliary indices for evaluating the acidity of fruits (Figure 3D and Figure 5C,D). Among the other three flavor contributors with TAV > 1, both GABA and Asn exhibited an astringent taste, while Arg imparted a bitter taste (Figure 5D) [32]. Among the astringent compounds, the TAV values of GABA and Asn in PG-treated fruits were significantly higher than those in other treatments, which may contribute to astringency in PG-treated fruits. Despite its astringent flavor, GABA plays pivotal roles in various physiological functions, including neurotransmission, blood pressure regulation, cellular signaling, and hormone regulation. Additionally, it offers protection against brain disorders, psychiatric conditions, cancer, and respiratory diseases [49]. Similarly, the researchers found in their Asn studies that extracellular Asn therapy could promote T cell activation in mice, enhancing their response to cancer cells and infections. Mice injected with melanoma cells showed stronger anti-tumor effects, with smaller tumors and higher survival rates [50]. Arg, as a functional amino acid, contributes to essential physiological processes such as promoting protein synthesis, cell division, wound healing, and hormone secretion [51]. In this study, Arg TAV in JJ-treated fruit was significantly higher at 4.86, 2.15 times higher than in CK. This finding is consistent with sensory evaluation results, suggesting Arg is one of the key contributors to fruit bitterness in the JJ treatment (Figure 1B and Figure 5D).

In summary, while high levels of GABA, Asn, and Arg contribute to the fruit’s astringency and bitterness, they also enhance its medicinal and health benefits. In addition, these results provide new pathways for obtaining plant-derived GABA, Asn, and Arg, which could be valuable for human health and wellness.

### 4.3. Interstocks Confer Different Antioxidant Capacity to Fruits by Influencing Phenolic Species and Content

Phenolic compounds, widely distributed secondary metabolites in fruits, are primarily represented by flavonoids and phenolic acids, known for their antioxidant potential [52]. In our study, we specifically examined 10 phenolic compounds enriched in citrus and detected a total of eight, with caffeic acid predominating among phenolic acids and naringin among flavonoids (Figure 6), consistent with prior research findings [35]. In the sinapic acid assay, we observed detection of sinapic acid exclusively in CJ- and JJ-treated fruits (Figure 6D). Sinapic acid, a derivative of cinnamic acid, exhibits diverse functions including potent antioxidant, anti-inflammatory, anticancer, hepatoprotective, cardioprotective, nephroprotective, and antimicrobial activities, potentially contributing to the unique antioxidant capacity observed in the two treatments of fruits [53]. Further evaluation of the fruit’s total antioxidant capacity revealed that the CJ and JJ treatments, which contained higher levels of sinapic acid, also exhibited higher DPPH values. Correlation analysis showed a significant positive correlation between DPPH and sinapic acid (r = 0.72), suggesting that sinapic acid plays a significant role in contributing to DPPH values (Figure 6 and Figure 7).

Combining the data from the evaluation of the three antioxidant methods, it was found that only the FRAP and APC results demonstrated good agreement and both were able to sensitively differentiate the antioxidant activity of the treatments. ABTS was unable to differentiate the antioxidant capacity of CK-, CJ- and JJ-treated fruits, and DPPH demonstrated similar limitations (Figure 7). The inability of the DPPH method to sensitively differentiate may be attributed to the antioxidant compounds’ structural conformation influencing interaction with DPPH radicals [54]. Conversely, the challenges faced by the ABTS method in sensitive differentiation could be linked to solution pH, as the ease of electron transfer in the reacting system is pH-dependent [55]. In this study, the pH values of CJ and JJ fruits were not significantly different, at 3.27 and 3.43, respectively. This provides direct evidence for the theory above (Figure 1I). Additionally, according to thermodynamic principles, compounds with redox potentials lower than ABTS^·+^ may react with free radicals, resulting in insignificant differences in the results [56]. Integrated analysis suggests that FRAP and APC are more suitable for evaluating antioxidant activity in this study. PG- and JJ-treated fruits exhibited a significantly higher antioxidant capacity, while the CK and CJ treatments showed relatively lower levels.

## 5. Conclusions

**Figure 9:**
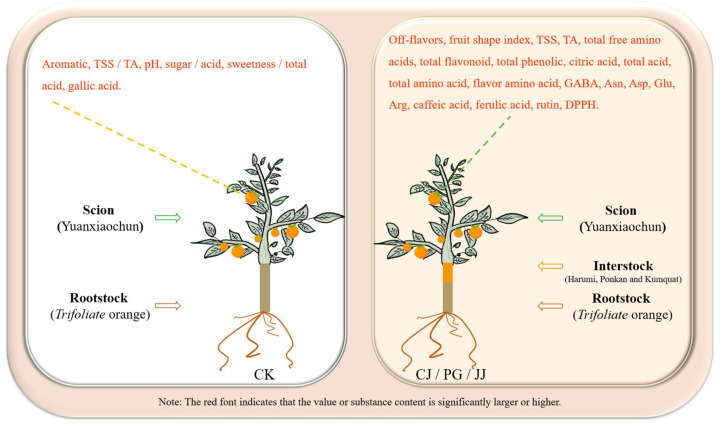
Comparison of effects of presence and absence of interstock on fruit quality of ‘Yuanxiaochun’.

In this study, we found that three different interstocks exerted a significant influence on the quality of ‘Yuanxiaochun’ fruits, particularly concerning sensory evaluation, the sugar/acid profile, and antioxidant capacity. Specifically, interstocks modulated the sugar and acid composition of ‘Yuanxiaochun’ fruits, thereby imparting varying degrees of sweetness and flavor, consequently impacting the fruits’ sensory attributes. Amino acids, notably GABA, Asn, Asp, Glu, and Arg, likely play pivotal roles in shaping the astringency, acidity, and bitterness of fruits. Regarding antioxidant capacity assessment, our results show that caffeic acid, naringin, and rutin all contribute significantly to the sum of eight phenols and overall antioxidant activity. Furthermore, in terms of antioxidant evaluation methods, we observed that FRAP and APC may be more appropriate than DPPH and ABTS assays for evaluating antioxidant activity of citrus fruits influenced by interstocks. Comprehensive analysis shows that compared with CK, interstock treatment significantly increased the levels of TSS, TA, total amino acids, flavonoids, total phenolics, and antioxidant capacity in ‘Yuanxiaochun’ fruit. However, it significantly decreased the fruit’s TSS/TA ratio, pH, sugar/acid ratio, and sweetness/total acid (Figure 9). Based on this observation, we conclude that interstock treatment increases the organic acid content in ‘Yuanxiaochun’ fruit, but it also enhances the levels of beneficial substances.

## Figures and Tables

**Table 1:** Test material treatment and abbreviations.

Treatments	Abbreviations
‘Yuanxiaochun’/‘*Trifoliate orange*’	CK
‘Yuanxiaochun’/‘Harumi’/‘*Trifoliate orange*’	CJ
‘Yuanxiaochun’/‘Ponkan’/‘*Trifoliate orange*’	PG
‘Yuanxiaochun’/‘Marumi Kumquat’/‘*Trifoliate orange*’	JJ

## Data Availability

All data used in this article can be found in this manuscript and in the Supplementary Materials.

## Supplementary Materials

The following supporting information can be downloaded at: https://www.mdpi.com/article/10.3390/antiox14101149/s1. Supplementary Table S1: Amino acid species and content of ‘Yuanxiaochun’ fruits grafted on different interstocks.
