# Supplementary material for: Effect of Different Interstocks on Fruit Quality, Amino Acids, and Antioxidant Capacity in ‘Yuanxiaochun’ Citrus
Source: Antioxidants (Basel). 2025 Sep 23;14(10):1149. doi: 10.3390/antiox14101149 (PMC12561795; doi:10.3390/antiox14101149)
Supplement: Supplementary file 1 [file antioxidants-14-01149-s001.zip › antioxidants-3786292-supplementary.pdf]

## Supplementary Table S1. Amino acid species and content of 'Yuanxiaochun' fruits grafted on different interstocks

| Sample name | Ala | GABA | Ser | Pro | Val | Thr | Ile | Leu | Asn | Orn | Asp | Gln | Lys | Glu | Met | His | Phe | Arg | Tyr | Trp | Gly | Hcy |
|---|---|---|---|---|---|---|---|---|---|---|---|---|---|---|---|---|---|---|---|---|---|---|
| CK1 | 81.26 | 204.53 | 113.73 | 542.31 | 13.86 | 23.91 | 5.96 | 7.15 | 295.81 | 23.41 | 137.98 | 87.13 | 28.96 | 72.06 | 4.84 | 9.38 | 15.51 | 484.39 | 8.65 | 4.98 | nd | nd |
| CK2 | 84.74 | 203.03 | 121.30 | 549.53 | 14.10 | 24.46 | 6.86 | 7.65 | 293.86 | 23.29 | 146.70 | 84.12 | 28.87 | 67.79 | 4.32 | 11.00 | 15.44 | 468.84 | 8.20 | 4.66 | nd | nd |
| CK3 | 80.23 | 207.47 | 122.44 | 540.44 | 13.88 | 25.69 | 6.57 | 7.56 | 285.45 | 23.61 | 146.85 | 87.06 | 29.28 | 70.44 | 4.49 | 9.55 | 15.57 | 472.49 | 8.28 | 5.13 | nd | nd |
| CJ1 | 213.13 | 217.42 | 188.55 | 1109.18 | 16.07 | 27.79 | 7.77 | 5.54 | 960.14 | 20.97 | 256.72 | 89.32 | 27.99 | 143.02 | 3.32 | 11.30 | 7.81 | 592.67 | 6.25 | 3.67 | nd | nd |
| CJ2 | 212.03 | 215.70 | 182.64 | 1079.93 | 16.00 | 25.56 | 7.11 | 5.20 | 964.97 | 20.41 | 263.99 | 88.03 | 27.86 | 136.08 | 3.18 | 10.68 | 8.51 | 576.72 | 5.93 | 3.81 | nd | nd |
| CJ3 | 212.42 | 217.57 | 177.56 | 1082.13 | 15.59 | 26.19 | 6.90 | 4.95 | 960.51 | 19.87 | 245.50 | 87.88 | 28.34 | 134.68 | 3.25 | 10.69 | 8.40 | 588.23 | 6.23 | 4.02 | nd | nd |
| PG1 | 320.07 | 295.44 | 237.57 | 1630.43 | 21.56 | 33.43 | 9.34 | 7.06 | 1456.65 | 28.89 | 357.68 | 111.29 | 40.25 | 188.39 | 4.72 | 15.01 | 9.45 | 912.12 | 6.81 | 5.08 | nd | nd |
| PG2 | 305.54 | 294.18 | 239.33 | 1596.72 | 21.69 | 33.20 | 8.62 | 7.16 | 1441.20 | 28.55 | 339.62 | 106.56 | 40.20 | 176.46 | 4.68 | 15.63 | 8.65 | 879.89 | 6.52 | 4.55 | nd | nd |
| PG3 | 294.67 | 275.23 | 226.53 | 1511.58 | 20.44 | 28.57 | 8.56 | 6.98 | 1345.73 | 26.40 | 341.01 | 98.76 | 35.25 | 164.87 | 3.97 | 14.34 | 8.84 | 828.46 | 6.16 | 4.51 | nd | nd |
| JJ1 | 264.77 | 238.42 | 217.03 | 1646.11 | 18.02 | 27.74 | 8.71 | 6.59 | 1321.82 | 38.20 | 267.57 | 133.24 | 42.92 | 143.90 | 3.80 | 16.31 | 13.70 | 1052.09 | 9.63 | 8.18 | nd | nd |
| JJ2 | 254.37 | 228.49 | 210.87 | 1563.68 | 18.13 | 29.21 | 7.56 | 6.41 | 1263.20 | 38.86 | 260.93 | 130.14 | 40.54 | 144.09 | 3.57 | 17.99 | 14.51 | 978.00 | 10.05 | 7.92 | nd | nd |
| JJ3 | 259.65 | 234.82 | 216.81 | 1583.39 | 19.75 | 29.54 | 8.32 | 7.02 | 1400.24 | 37.84 | 278.58 | 130.51 | 42.39 | 139.75 | 3.72 | 17.92 | 14.82 | 1028.17 | 10.70 | 8.56 | nd | nd |

Note: 'nd' indicates not detected.
